# Exploring women’s thoughts on self-weighing during pregnancy: results of the Self-Weighing in Pregnancy: Experiences (SWIPE) study

**DOI:** 10.1186/s12884-021-03636-5

**Published:** 2021-02-20

**Authors:** Anne E. Ferrey, Nerys M. Astbury, Yvonne Kenworthy, Lucy Mackillop, Kerstin Frie, Susan A. Jebb

**Affiliations:** 1 Nuffield Department of Primary Care Health Sciences, University of Oxford, Radcliffe Primary Care Building, Radcliffe Observatory Quarter, Woodstock Road, Oxford, OX2 6GG UK; 2 NIHR Oxford Biomedical Research Centre, Oxford, UK; 3 Nuffield Department of Women’s and Reproductive Health, University of Oxford, Oxford, UK; 4 Oxford University Hospitals NHS Foundation Trust, Oxford, UK

**Keywords:** Gestational weight gain, Self-weighing, Self-monitoring, Self-regulation

## Abstract

**Background:**

Excess gestational weight gain is common and an important risk factor for adverse pregnancy outcomes. Regular weighing can be used to assess and manage weight gain, but NICE guidelines do not recommend routine weighing during antenatal care. Trials that have tested the effectiveness of self-weighing to manage GWG have been unsuccesful in engaging women in regular self-weighing, although the reasons for lack of engagement are not fully understood. This study aimed to understand why this lack of engagement occurred by exploring the naturally occurring thoughts and feelings of pregnant women (9 to 15 weeks gestational age) who were asked to weigh themselves at home.

**Methods:**

Twenty-five women were recruited to take part. Participants completed short questionnaires at their first-trimester and 20-week scans. After recruitment, participants were asked to weigh themselves at roughly the same time each week for 8 weeks. Whilst they weighed themselves they were asked to audio-record their current weight and describe any thoughts or feelings that occurred as they weighed themselves. These audio recordings were then sent to researchers using a secure messaging service.

**Results:**

Most of the recruited women (56%) were unaware of guidelines for gestational weight gain, and only 40% could identify the ideal rate of GWG for their BMI group. Thematic analysis of the think-aloud recordings resulted in three main themes: “understanding weight gain in pregnancy”, “taking action to prevent weight gain” and “reactions to self-weighing”. Overall, there was a relatively positive response to self-weighing and some participants used self-weighing to reflect on the reasons for weight gain and plan actions they could take to avoid excess gain. Negative emotional responses tended to be related to a lack of guidance about what level of weight gain or loss was “healthy”, or to other worries about the pregnancy. Of the women recruited who submitted at least one think aloud recording (n 10), 80% found self-weighing to be useful, and said they would likely continue to self-weigh at home.

**Conclusions:**

Women had complex emotions about self-weighing during pregnancy but overall found it useful, suggesting it could be encouraged as part of self-regulatory interventions to control GWG. Clear guidelines about appropriate gestational weight gain could help to reduce anxiety.

**Trial registration:**

The study was prospectively registered with ISRCTN ISRCTN10035244.

**Supplementary Information:**

The online version contains supplementary material available at 10.1186/s12884-021-03636-5.

## Background

Gaining too much weight during pregnancy, also called excess gestational weight gain (GWG), is a major modifiable risk factor for several adverse pregnancy outcomes. These include having a large for gestational age infant, macrosomia, caesarean section delivery, gestational diabetes mellitus (GDM), preeclampsia, postpartum weight retention and obesity in the infant [9, 10, 17, 23]. Nonetheless, excess GWG is relatively common, with an estimated 40–60% of women gaining more weight than recommended in the revised 2009 IOM guidelines [8, 11]. The increasing problem of obesity and excess GWG in maternity care has led to national clinical recommendations advising the development of interventions to manage GWG to improve pregnancy outcomes [11, 15]. In turn, numerous interventions have been developed to help attenuate GWG, and have been tested in several clinical trials [5, 18, 19]. These interventions have mainly involved intensive antenatal contact focusing on providing information to women on changing diet and increasing physical activity, with limited success [21].

Techniques that have proved to be helpful for preventing weight regain in women with overweight might also work to prevent excess gestational weight gain (GWG) in pregnancy. One technique that is consistently associated with successful weight gain prevention is regular self-weighing [22]. The rationale is that regular self-weighing provides feedback on weight changes and thus allows the individual to make timely adjustments to prevent weight gain [2, 13]. This is hypothesised to occur via a process of self-regulation. First, the individual compares their current weight to a previously-set goal weight, and then uses this information to plan and implement changes to their behaviour to reduce discrepancies [13]. The regular weighing further allows individuals to evaluate the effect of their past behaviour, enabling them to learn which actions they can take to manage their weight.

However, several randomised controlled trials (RCTs) have failed to demonstrate that routine antenatal and/or self-weighing interventions attenuate GWG and improve perinatal outcomes [3, 12, 14]. A recent RCT in which midwives weighed women during routine antenatal appointments, set targets for gestational weight gain, and advised women to weigh themselves weekly, did not show evidence of reducing the proportion of women who had excessive GWG between 10 and 14 and 38 weeks gestation [4]. However, the authors note that midwives only recorded weight on 57% occasions and that only half of the participants weighed themselves on 5 or more occasions and a third not at all or only once. The lack of engagement with self-weighing is likely to have contributed to the lack of effect of the intervention on gestational weight gain, but the reasons why women did not engage with regular self-weighing at home are not fully understood.

International practices on weighing during pregnancy  vary considerably, although a recent survey of 53 countries found that 81% had a policy to monitor GWG, and of these, 62% provided recommendations for healthy GWG [20]. In the USA, for example, regular antenatal weight monitoring is encouraged, and gestational weight gain ranges are recommended based on early pregnancy BMI [11, 16]. However, in the UK, NICE clinical guidelines state that women should be weighed at booking, but there is clear instruction that they should not be routinely weighed repeatedly during pregnancy unless clinical management can be influenced or nutrition is a concern [15].

As a result, for the vast majority of women in the UK, unless they decide to regularly monitor their weight of their own accord, they are not routinely weighed during antenatal appointments and are not advised to weigh themselves. However, in order for women to employ self-regulation strategies that may help them regulate GWG, they must either be weighed by a healthcare professional during routine antenatal visits or they must self-weigh regularly during pregnancy.

The aim of this study was to explore the naturally occurring thoughts and feelings of pregnant women asked to weigh themselves at home during weeks 9–20 of pregnancy. We aimed to identify any barriers and/or facilitators to self-weighing which may aid the future development of interventions to manage GWG using a self-regulatory approach.

## Methods

This was a prospective cohort study using a qualitative think-aloud methodology, conducted at Oxford University Hospitals NHS Foundation Trust. The study protocol and procedures were reviewed and approved by Health Research Authority (HRA) North East – Newcastle & North Tyneside 2 Research Ethics Committee (Ref:19/NE/0083) and all participants provided written consent to participate before they were enrolled on the study. Participants also agreed to allow any individual pseudonymised quotations to be published.

### Participants

All women who schedule antenatal appointments at the Women’s Centre at Oxford University Hospitals NHS Foundation trust provide “consent to be approached” to take part in a research study. Potentially eligible pregnant women between 9 and 15 gestational weeks with a first trimester BMI (Body Mass Index) < 25 kg/m^2^ attending their first trimester scan at the Women’s Health clinic at the John Radcliffe Hospital in Oxford, England, were identified by a member of the clinical care team. One of the researchers introduced themselves to these potentially eligible women and verbally explained who they where and why they were approaching them, and provided an information leaflet which summarised what was involved in taking part in this study. If women indicated that were interested in taking part in the study they were provided with a copy of the participant information sheet to read whilst they were waiting for their scan. Once they exited the scan room, those who were still interested in taking part met with one of the researchers in a semi-private part of the waiting area where the researcher answered any questions they had and obtained written informed consent to take part.

### Procedure

Eligible women who provided consent to take part in the study were asked weigh themselves at home, at approximately the same time each week for eight consecutive weeks following the first trimester scan (baseline). They were asked to use their home weighing scales, or were provided with scales for the duration of the study. During the process of weighing themselves they were asked to audio record themselves using the voice memo function of their mobile phone. In each recording they were asked to state their current weight and then to think out aloud about any thoughts or feelings that naturally occurred as they weighed themselves [6, 7]. Once they had finished the process they were asked to send the audio recording file to the research team using a secure messaging service. A baseline questionnaire was provided to the participants to self-complete at the first trimester scan and a similar follow-up questionnaire was provided to participants to complete either at the 20 week scan appointment, or to post with a postage paid return envelope if the 20 week scan appointment was scheduled before the end of the think-aloud recordings. All participants were provided with a £50 gift voucher for taking part.

### Data analysis

Think-aloud recordings were transcribed verbatim. An inductive thematic analysis (Braun & Clarke, 2012) using NVivo software (Version 12) identified codes from recurring topics. Codes were aggregated into themes using an OSOP (‘One Sheet of Paper’) analysis [24].

## Results

### Sample characteristics

Twenty-five women were recruited to the study with an average age of 29 years.

Of the 25 women that were recruited to the study, only 13 (25%) provided any recordings. Two had complications with their pregnancies identified after the 12 week scan and were not followed up. Eleven participants (44%) recorded at least 5 weeks of think-aloud interview data. Only four (16%) submitted the full 8 weeks of think-aloud recordings that were requested. Follow-up questionnaire data were received from twelve participants (48%), but one of the respondents did not submit any think-aloud recordings (see Supplementary Material for baseline and follow-up questionnaires).

### Baseline questionnaire data

The baseline questionnaire data showed that the majority (56%) of women recruited to the study (n 14) said they had never heard of the Institute of Medicine guidelines for weight gain during pregnancy. Nobody answered that they knew the guidelines well. Of the women who indicated that they had heard of the guidelines, 40% (n 10) correctly identified the recommended amount of weight gain for themselves.

The majority of women (56%) were ambivalent about self-weighing before they entered the study, with smaller proportions either liking it/ liking it a lot (16%), or disliking it /disliking it a lot (32%).

All but one woman (n 24) indicated that they had been weighed by a healthcare professional since they had discovered that they were pregnant and 40% of women had been given advice on weight gain during pregnancy.

Of those who submitted at least one think-aloud recording (n13), 69% reported having weighed themselves at some point throughout pregnancy (Table 1), in comparison those who did not submit a recording (n 9) (after removing those who had complications with the pregnancy) 90% reported having weighed themselves at some point during their pregnancy.

#### Think-aloud data

**Fig. 1:**
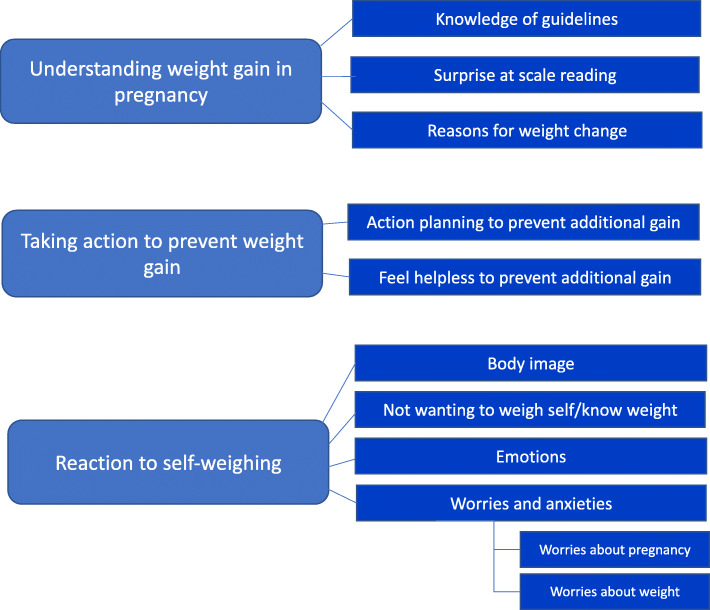
Themes and sub-themes emerging ftom the thematic analysis of think aloud recordings of pregnact women asked to regulary self-weigh themselves during the first half of pregnancy

Three themes emerged from the thematic analysis of the think aloud recordings (Fig. 1). These were “understanding weight gain in pregnancy”, “taking action to prevent weight gain” and “reactions to self-weighing”. These themes were interlinked and each contained a number of subthemes.

**Table 1:** Self-reported frequency of self-weighing of women enrolled in the SWIPE study at baseline (*n* 25)

	Daily	Weekly	Monthly	Less frequently than monthly	Not at all
	n (%)	n (%)	n (%)	n (%)	n (%)
How often did you weigh yourself when you were not pregnant?	2 (4)	7 (28)	5 (20)	7 (28)	4 (16)
How often have you weighed yourself since you found out you were pregnant?	1 (4)	7 (28)	9 (36)	3 (12)	5 (20)

#### Understanding weight gain in pregnancy

“Understanding weight gain in pregnancy” could be broken down into three subthemes (Fig. 1). The first was “knowledge of guidelines”. Participants described confusion regarding how much weight they should be gaining at this stage of pregnancy.

*“I'd quite like to know how much weight is a normal amount of weight to put on* … *I'm a bit confused as to what I'm supposed to be, what's a healthy weight to put on I don't wanna put on too much but also I don't wanna not eat enough.”* (Abby)

Some participants specifically commented that they would like to have been given guidelines about weight gain in pregnancy as they were *“not sure how much of the weight is pregnancy and how much is just weight gain” (Abby)*

*“I do wish that … I did have an idea of how much weight to put on during my pregnancy … I don't feel there is many guidelines out there … perhaps even like a leaflet could be helpful just sort of outlining, you know, how much to put on during … the first trimester and second and so on.”* (Holly)

In general, participants mentioned that they thought they should gain weight during pregnancy; although as above, they were not clear on how much or how fast this should be gained. One participant characterised her gain at 20 weeks as “*putting on weight quite nicely” (Kathleen).*

*“So I’ve gained another bit of weight this week, which I’m quite pleased about, makes me feel happy … I do think it’s really good to gain weight during your pregnancy … ” (Leah)*

One person actually spoke to the midwife because she had *not* put on any weight in early pregnancy, but the midwife “*said it’s not much to worry about, so I’m happy with that*” (Isobel).

Many women commented on their surprise when their expectations of weight gain in pregnancy did not align with reality. Many participants seemed to expect to gain weight each week and were surprised when this was not the case. Kathleen said she was “*gaining weight slowly … I thought I probably would have put on more weight by now but obviously not.”* However, other participants were surprised when they had gained more than expected. “*I’ve put on a lot more weight than I had hoped” (Deanna).*

After expressing surprise, many participants then began to think out aloud about reasons for their unexpected scale reading. A sub-theme here related to participants’ views on the reasons why they had gained or lost weight – due to their own behaviours or factors outside their control.

*I seem to be a bit lighter than last week … I'm not sure if that's due to the fact that I was poorly as I had a fibroid so my stomach was bloated so maybe that's had a factor in it, I'm not sure. I'm very surprised by that, I thought I would have put a little bit of weight on” (Leah).*

It was very common for participants to describe their actions in the previous week and relate these to the number they saw on the scale. This was done spontaneously, as participants were not asked to record any specific details about why they might have gained or lost weight, but shows reflection about the reasons for the change.

*I've put on a lot more weight than I had hoped to … I think it might be down to I'm snacking a lot more because I've given up smoking.(Deanna)*

#### Taking action to prevent weight gain

A few women spontaneously took the next step in the self-regulation process and developed action plans to control their rate of weight gain in the future. They described steps they planned to take to prevent excessive weight gain. These ranged from quite vague (“*I’m gonna try and up my daily exercise from now on”* [Deanna]) to quite concrete action plans (“*more home cooked food so really trying to cut out on the processed food” [Holly]; “I’ve been doing more cycling and exercise” [Michaela]).* Other concrete actions included taking iron liquid (“*it’s helping me get out and do a bit of exercise as well” [Isobel]),* avoiding snacking on sweets and increasing intake of fruit and vegetables. This suggests that most women felt they had agency over their weight gain and felt they could take action to change their weight gain trajectory. However, some women felt they were unable to have any influence on their rate of weight gain due to side effects of the pregnancy.

*“Today I am 14 weeks pregnant and my weight is 98 kg and 4 gram. It's going up very fast but there is nothing much I can do at the moment about it because of sickness and tiredness.” (Eva)*

#### Reaction to self-weighing

We particularly wanted to investigate participants’ thoughts and feelings about the process of self-weighing. During recruitment, participants were told “we want to know what you think about when you are weighing yourself”. No more specific instructions were provided, as we wanted participants to relay their thoughts as they came up. This theme relates to participants’ thoughts and emotions regarding the process of self-weighing and seeing the number on the scale. A number of sub-themes were identified: not wanting to weigh oneself or know one’s weight; body image; feelings about weight and self-weighing; and being worried, anxious or stressed in relation to pregnancy or self-weighing.

Participants’ desire to weigh themselves in pregnancy varied. Overall, many of the think-aloud recordings were brief and generally positive – despite being unaware of GWG guidelines, many respondents were content with the process of self-weighing and with the results on the scale. “*Pretty happy, I mean no major weight gain but pretty happy*” (Kathleen). “*I’m still gaining weight and I’m gaining it slowly so I’m really happy with that progress*.” (Leah).

However, a subset of participants did not want to self-weigh or to know their weight. Although all were fully consented and agreed to weigh themselves for the duration of the study, many women did not complete the task. The reasons for this drop-out are unknown. Among women who did submit think-aloud recordings some women clearly found the task uncomfortable. Generally, this was related to knowing or suspecting that they had put on more weight than recommended. Sometimes this was temporary, for instance during a week in which they felt they had not acted as healthily as they should. “*Okay I’m not looking forward to weighing myself today … I think I’ve eaten quite a lot this week so no doubt I’ve probably put on quite a bit of weight*” (Holly). In other cases, this seemed to be a more general wish to avoid thinking about their weight.

*“I was dreading getting on the scales because when I saw my weight in the hospital it was quite a bit higher than I thought …* ”(Bella)

Other participants mentioned feeling nervous about weighing themselves or embarrassed about reporting their weight. *“Feeling a bit nervous today weighing myself … I’m sort of losing control of the weight a bit.”* (Abby) “*Have to be honest I had to resist the temptation to make an excuse for why my weight’s gone up or potentially even tell you that I haven’t put on as much as I have, I feel quite embarrassed.*” (Bella).

Self-weighing led some participants to reflect on their feelings about their body shape and weight. In some cases they described positive thoughts: “*I am 10 stone 13.4 lbs, I think that’s a reasonable weight for my height and shape … I feel quite comfortable, I don’t feel obese or anything”* (Leah). In other cases, participants had a less positive conception of their size and shape. *“I’m a little bit sort of worried at the size of my tummy at the moment, I know it’s my second baby but I’m just a little bit conscious of it” (Faith).*

Some participants did mention general worries related to pregnancy. “*My scan’s tomorrow so I’m looking forward to having that, I have been feeling a bit anxious about that so hopefully that will all go like clockwork and be fine.”* (Holly).

Anxieties related to pregnancy were sometimes discussed as a reason for *not* thinking about or being worried about their weight. Bella, for example, had been told that she had very low levels of PAPP-A and this overshadowed any thoughts of her weight gain during pregnancy.

“*My mind is so far away from my weight right now it's, I don't really, I'm not eating that well but, you know, I've had two phone calls from the midwives at the [hospital] and my PAPP-A is really low, it's so distressing I don't know what I can do apart from take aspirin but what they're saying is so distressing that actually my weight couldn't be further from my mind. The anxiety [um] that's coming from that means my weight is, is just not a concern right now, maybe it should be but it's not*.” (Bella)

Other consequences of self-weighing included worrying about weight changes – both gains and losses. Sudden or perceived excessive weight gain caused concern, particularly in relation to the pregnancy: “*I’m pretty mad that I was already pretty heavy before getting pregnant and now whenever I see this weight I just get scared that this is gonna have [um] bad implications for my baby” (Julia).* However, women also worried about weight loss. Most women described having expected to gain weight in pregnancy, so any losses stirred up anxiety. “*I feel a lot better because I haven’t lost weight again which I was quite worried about”* (Caroline). Often both concerns were related by the same participant over time. “*I didn’t like the fact that I was putting on a lot of weight so fast but it’s quite a bit to lose in a week when I’m supposed to be growing a human, so I feel a bit bittersweet about it*.” (Caroline).

*“I mean … at the same time that I wanna know my weight and that everything is progressing it's just giving me all sorts of questions and anxiety about it because at the beginning I was worried that I was too heavy and now that I've only put on half a kilo … in two weeks I'm like is this normal, is this okay … so I'm actually feeling quite stressed about my weight for totally different reasons than at the very beginning [um] which I don't think is particularly helpful right now.” (Julia)*

One participant was “terrified” at losing two kilograms in a week and commented, “*this weighing thing … has been super stressful … you want my thoughts on weighing … there needs to be support for you weighing yourself weekly to understand the results or what they could at least mean.” (Julia).*

However, others were quite sanguine about fluctuations in weight. *“15 weeks and I’ve gained a little which obviously during Christmas I’m not really worried about, I keep going up and down at the moment so I’m not overly worried about gaining a few pounds”* (Kathleen).

### Follow-up questionnaire data

The follow-up questionnaire asked how useful participants found self-weighing to be. This data was collected only from participants who completed at least one think-aloud recording (n 10). Eighty percent (n 8) of these participants found self-weighing to be useful, and a further 80 % of participants who completed the questionnaire said they would “probably” or “definitely” continue to self-weigh after the end of the study. Participants liked “keeping track of how much weight they were gaining” and “feeling in control of how much weight they were gaining”. Half said there was nothing they particularly disliked about weighing themselves, although 30% indicated that self-weighing led to worries that they were not gaining the “correct” amount.

Participants' written comments on the follow-up questionnaire were also revealing. These captured participants’ overall thoughts about self-weighing, in contrast to the in-the-moment data collected during think-aloud recordings. Participants noted what they found useful about self-weighing: “*I think weighing myself helped to me to keep myself checking what I was eating*”; “*made me think about what and how much I was eating and how it would affect both me and my baby*”; “*helped me to actually think about how I felt*”. Negative comments focussed on the perceived lack of guidelines for gestational weight gain: “*I felt like I didn’t know if it was a “good“ or “normal“ amount of gain.”*

*“I don't think my issue with weighing myself is to do with the weight itself but more that I was doing it unsupported. This is a risky pregnancy for me and not being able to contextualise the weight gain and loss didn't help*”.

*“Weighing yourself and seeing that you have lost weight actually caused a bit of concern. I worried that the baby wasn't okay.”*

Perceived lack of information about expected weight gain could also be perceived positively, however – one participant said, “*I was expecting to pile on weight so I liked that when I weighed myself, for the most part, I felt quite positive.”*

## Discussion

This study used mixed methods, including in-the-moment think-aloud recordings of participants’ thoughts during weighing, and quantitative survey feedback. It aimed to shed light on how women feel about weighing themselves during pregnancy and whether they use this information to self-regulate in order to prevent excess weight gain. We found that most women were unaware of guidelines for weight gain during pregnancy and did not know how much weight they were recommended to gain. Women were asked to record their thoughts while weighing themselves each week, and three main themes emerged from the analysis of these recordings: troubles with understanding weight gain in pregnancy, descriptions of actions they took to prevent weight gain, and emotional reactions to self-weighing. In line with the questionnaire data, many women did not understand how much weight they should be gaining and were surprised by the numbers on the scale. Many commented that they would like to be given more information about healthy weight gain and GWG guidelines. Overall, the participants who submitted think-aloud recordings held a range of views at the start about self-weighing. Hence, participants did not complete the study solely because they already enjoyed self-weighing.

Women in this study speculated about reasons for their weight gain or loss, and some described action plans to mitigate excess weight gain. This reflection is a key step towards the evaluation of previous behaviour: in order to identify which actions are helpful and which ones are to be avoided to prevent excessive weight gain in pregnancy, a person must first be cognisant of the actions they have previously taken that might have affected their weight. Based on the reflection and evaluation process, individuals can then continue with those behaviours that have brought them success in the past, and additionally try out new strategies to ensure they reach their goals.

Although there was a relatively positive response overall to the idea of self-weighing, some women did not want to weigh themselves and felt they would be embarrassed about their weight gain. Lack of awareness of the weight gain guidelines meant that weighing could be stressful as they did not understand whether a large gain or loss was problematic, and other anxieties about the pregnancy could interfere with thinking about GWG at all. Women largely found self-weighing to be useful and said it helped them to think about how their eating habits influenced their GWG. However, some noted that the lack of guidance on weight gain led them to feel unsupported.

### Strengths and limitations

This study was the first to capture the thoughts of women who were pregnant at the moment of self-weighing. It captured women’s reflections on the process of weighing and on their weight and its relationship to their pregnancy. The qualitative data is rich and gives valuable insights for future interventions. However, limitations include the small sample size and the fact that data was collected only from women with a pre-pregnancy BMI between 25 and 30, who were willing to try weighing themselves regularly during the first half of their pregnancy.

### Implications

Previous trials that incorporated self-weighing as a strategy to reduce excess GWG have had limited success. This may be due to a lack of understanding of the emotions caused by self-weighing during pregnancy. It has been assumed that women are willing to weigh themselves regularly, but previous adherence data and our qualitative findings suggest that this is not always the case. Our results show that women express complicated emotions related to self-weighing and weight gain or loss during pregnancy. These were particularly difficult when women were concerned or anxious about some aspect of the pregnancy, or felt they did not understand whether they were gaining weight at the “correct” rate because they were not aware of the guidelines. Nonetheless, most women felt broadly positive about self-weighing.

Pregnancy already causes a great deal of stress for many expectant mothers. Self-weighing is not currently recommended for those who are pregnant, as there are concerns this may add additional stress. These worries could link to the first theme “*Understanding weight gain in pregnancy*”, in that women were worried that they might not be gaining weight appropriately because they didn’t understand how much they should be gaining, and this is consistent with data from the baseline questionnaires revealing a lack of awareness concerning the weight gain guidelines prior to being enrolled in the study.

Our results suggest that self-weighing could be made more helpful and less worrisome if women were provided with guidelines on appropriate GWG. However there is a need to develop UK guidelines and the paucity of data on weight gain in pregnancy for women in the UK is a barrier.

Even when people self-monitor their weight there is no guarantee that it will act as an effective intervention to prevent excess GWG. Weight loss research suggests that self-weighing on its own is unlikely to be sufficient to trigger a self-regulation response in everyone [6, 7]. Individuals may therefore have to be guided through self-regulation after self-weighing to make this strategy effective. Previous trials on self-weighing in pregnancy have failed to address this [3, 12, 14]. Guidance through self-regulation should include the provision of feedback on healthy GWG to help contextualise weight changes, support in evaluating previous behaviour, and prompts to form action plans based on identified weight changes. Weight loss research taking this approach of guided self-regulation in non-pregnant participants has shown promising results in the short-term [6, 7].

There is an important distinction between previous trials that asked women to self-weigh and the practice of routinely weighing women by a healthcare professional during antenatal visits. However, the experiences of the women in this study who self-weighed themselves at home during the first half of pregnancy suggest that it may be time to consider re-introdoucing regular weighing during routine antenatal appointments [1]. A re-introduction of the practice would give health professionals the opportunity to discuss GWG and a healthy rate of weight gain which may alleviate some of the stress and anxiety the women in this study experienced about their weight, and which is plausibly occurring more generally among women who weigh themselves during pregnancy and perhaps also those who fear weighing themselves. Routinely collecting information on weight change during pregnancy would generate a vast amount of data on weight gain during pregnancy, which could be used to establish UK specific appropriate GWG ranges [20].

## Conclusions

Women had complex emotions about self-weighing during pregnancy but overall found it useful. Regular weighing by a midwife during routine appointments, clear guidelines about appropriate gestational weight gain and incorporation of self-weighing as part of a broader self-regulatory intervention may help to reduce anxiety and could form the basis of interventions to help women to avoid excess GWG.

## Supplementary Information

**Additional file 1. **Baseline questionaire.

## Data Availability

The datasets generated and/or analysed during the current study are not publicly available as they may contain sensitive and identifiable information, but are available upon reasonable request (information.guardian@phc.ox.ac.uk).

## Abbreviations

BMI: Body Mass Index
GWG: Gestational weight gain
IOM: Institute of Medicine
NICE: The National Institute for Health and Care Excellence
RCT: Randomised Controlled Trial
