# Supplementary material for: Exploring women’s thoughts on self-weighing during pregnancy: results of the Self-Weighing in Pregnancy: Experiences (SWIPE) study
Source: BMC Pregnancy Childbirth. 2021 Feb 20;21:154. doi: 10.1186/s12884-021-03636-5 (PMC7897370; doi:10.1186/s12884-021-03636-5)
Supplement: Supplementary file 1 — Additional file 1. Baseline questionaire. [file 12884_2021_3636_MOESM1_ESM.pdf]

PARTICIPANT ID: ☐☐☐☐

BASELINE QUESTIONAIRE

1. Have you been weighted by a healthcare professional at all since you found out you were pregnant?
   - ☐ Yes
   - ☐ No
   - ☐ I can't remember

2. Have you been given any specific advice about weight and weight gain during pregnancy?
   - ☐ Yes
   - ☐ No
   - ☐ I can't remember

If you answered *YES* to Q2, How did you feel about this advice?

3. How often did you weigh yourself usually- when you're not pregnant?
   - ☐ Daily
   - ☐ Weekly
   - ☐ Monthly
   - ☐ Less frequent than monthly
   - ☐ Not at all

4. How often have you weighed yourself since you found out you were pregnant?
   - ☐ Daily
   - ☐ Weekly
   - ☐ Monthly
   - ☐ Less frequent than monthly
   - ☐ Not at all

5. How do you feel about weighing yourself? (Likert scale from dislike it a lot to like it a lot)
   - ☐ I like it a lot
   - ☐ I like it
   - ☐ I neither like it or dislike it
   - ☐ I dislike it
   - ☐ I dislike it a lot

6. Are you aware of the Institute of Medicine (IOM) guidelines for weight gain during pregnancy?
   - ☐ Yes, I know them well
   - ☐ Yes, I've heard of them
   - ☐ I have never heard of the IOM guidelines for gestational weight gain

7. How much weight do you think is recommended for you to gain during your pregnancy?
   - ☐ More than 20 kg (approximately 3 stone)
   - ☐ Between 12 and 20 kg (approximately between 2 and 3 stone)
   - ☐ Between 10 and 15 kg (approximately between 1 ½ and 2 stone)
   - ☐ Between 5 and 10 kg (approximately between 1 and 1 ½ stone)
   - ☐ Under 10 kg (approximatley 1 ½ stone)

PARTICIPANT ID: ☐☐☐☐

FOLLOW-UP QUESTIONNAIRE

1. How useful did you find weighing yourself throughout the study?
   - ☐ I found it very useful
   - ☐ I found it useful
   - ☐ I neither found it useful or not useful
   - ☐ I found it not useful
   - ☐ I found it not useful at all

2. Was there anything that you particularly liked about weighing yourself?
   - ☐ There was nothing that I particularly liked about weighing myself
   - ☐ I liked keeping track of how much weight I was gaining
   - ☐ I liked feeling I was in control of how much weight I was gaining
   - ☐ I liked feeling re-assured that I was gaining the 'correct' amount of weight
   - ☐ I liked the knowledge that my baby was growing
   - ☐ Other (please specify):

3. Was there anything that you particularly disliked about weighing yourself?
   - ☐ There was nothing I particularly disliked about weighing myself
   - ☐ I disliked seeing how much I weighed because it made me feel bad
   - ☐ I disliked weighing myself because I worried I was not gaining the 'correct' amount
   - ☐ I disliked weighing myself because I felt like I was eating too much/too little
   - ☐ I disliked
   - ☐ Other (please specify)

4. Has thinking-aloud affected the way you thought or felt during weighing?
   - ☐ Yes
   - ☐ No
   - ☐ I don't know

   If you answered yes to Q4- please choose any options that describe the way the think aloud process has affected the way you think about weighing yourself during pregnancy?

5. Do you think you will continue weighing yourself throughout the rest of the pregnancy?

- ☐ Definitely
- ☐ Very probably
- ☐ Probably
- ☐ Possibly
- ☐ Probably not
- ☐ Definitely not
